# Supplementary material for: A Qualitative Study on the Use of the Hospital Safety Index and the Formulation of Recommendations for Future Adaptations
Source: Int J Environ Res Public Health. 2023 Mar 12;20(6):4985. doi: 10.3390/ijerph20064985 (PMC10049632; doi:10.3390/ijerph20064985)
Supplement: Supplementary file 1 [file ijerph-20-04985-s001.zip › ijerph-2272004-supplementary.pdf]

A qualitative study on the use of the hospital safety index and the formulation of recommendations for future adaptations.

Interview Guide

Introduction of interviewees and the research team.

Explanation of study design and objective.

Request for oral informed consent.

Request for consent to record the interview.

1. General demographic questions.

- What is your professional background?
- How many years of experience do you have in your role?
- In what year did you first use the HSI?
- Which HSI version did you use (2008 vs. 2015)?
- What type of hospital the tool was used in (big, small / private, public…)?

2. Why have you chosen the HSI as a measurement tool instead of others, and what led to this choice?

3. Before starting data collection, what measures/strategies have you put in place to ensure the proper use of the tool?

- Did you attend any training?
- Did you read some documents/instructions?
- Did you implement any modifications to the tool?
- Did you meet/consult any expert in the field?

4. Can you explain step-by-step how did you used the tool for data collection?

- Did you form a team? If so, what were its members?
- What data collection methodologies did you use (visual inspections, interviews, etc.)?

5. While collecting data using the HSI, what are the main difficulties you encountered and how you managed to overcome them?

- If any, what do you think the tool did not capture when assessing preparedness?
- Did you find it easy to apply the HSI to your cultural context?
- How long did it take for you to complete data collection using the HSI?

6. Do you have any suggestions or recommendations for future usage or for future adaptations of the tool?

- Overall, how did you find the tool?
- What could make hospitals and researchers use the tool more effectively?
- Do you have any operative advice for people deciding to use the HSI for the first time?

7. Have you used the HSI again recently or do you know others who have used/are using it?

8. Do you have anything you want to add?

Close the interview, greetings and stop the recording.
